# A personality trait contributes to the occurrence of postoperative delirium: a prospective study

**DOI:** 10.1186/s12888-016-1079-z

**Published:** 2016-11-03

**Authors:** Jung Eun Shin, Sunghyon Kyeong, Jong-Seok Lee, Jin Young Park, Woo Suk Lee, Jae-Jin Kim, Kyu Hyun Yang

**Affiliations:** 1 Institute of Behavioral Science in Medicine, Yonsei University College of Medicine, Seoul, Republic of Korea; 2 Severance Biomedical Science Institute, Yonsei University College of Medicine, Seoul, Republic of Korea; 3 Department of Anesthesia and Pain Medicine, Yonsei University College of Medicine, Seoul, Republic of Korea; 4 Department of Psychiatry, Yonsei University College of Medicine, Seoul, Republic of Korea; 5 Department of Orthopedic Surgery, Yonsei University College of Medicine, Seoul, Republic of Korea; 6 Department of Psychiatry, Gangnam Severance Hospital, 211 Eonju-ro, Gangnam-gu, 06273 Seoul, Republic of Korea; 7 Department of Orthopedic Surgery, Gangnam Severance Hospital, 211 Eonju-ro, Gangnam-gu, 06273 Seoul, Republic of Korea

**Keywords:** Delirium, Risk factor, Personality, Logistic regression, Topological data analysis

## Abstract

**Background:**

Although various physical risk factors for delirium have been identified, the effect of psychological aspects is currently unknown. This study aimed to examine psychological risk factors for postoperative delirium and to identify hidden subgroups of delirium in clinical and psychological feature space.

**Methods:**

Among 200 patients with hip fracture, 78 elderly patients were prospectively evaluated for clinical and psychological assessments before surgery. As delirium was assessed from the next day to the 7th day after surgery, postoperative delirium was found in 40 patients, but not in 38 patients. Univariate and multivariate logistic regression analyses were used to explore risk factors for postoperative delirium. Phenotypic subgroups of delirium were assessed using Topological Data Analysis, in which the significant risk factors were used for evaluating filter and distance metrics.

**Results:**

Mini-Mental State Examination, neuroticism, conscientiousness, and regional anesthesia were identified as a predictive risk factor for postoperative delirium. The filter metric showed significant negative correlations with nutrition-related factors such as total protein and albumin. When filter metric and Euclidean distances were entered, delirious patients were bifurcated as a function of personality traits and anesthesia method in the patient-patient network.

**Conclusions:**

A personality trait of neuroticism and conscientiousness may predispose elderly patients to postoperative delirium and this influence may be amplified by regional anesthesia. This study verifies the contribution of psychological risk factors to delirium and provides new insight for complex etiologies of delirium by mapping various clinical variables in the topological space.

**Electronic supplementary material:**

The online version of this article (doi:10.1186/s12888-016-1079-z) contains supplementary material, which is available to authorized users.

## Background

Delirium is an acute confusional state that is characterized by sudden alteration and fluctuation of mental status, inattention, and disorganized thinking. Although delirium mostly disappears within a few days, it can result in a delayed medical recovery, prolonged hospitalization, and increased costs [1]. For example, early mobilization, which is critical in patients with hip fracture, can be hampered until the recovery of delirium. Delirium is known to be reversible, but recent reports have suggested that the cognitive decline experienced during delirium lasts after recovery [2, 3]. Various abnormal physical conditions have been reported to be associated with delirium, though the pathophysiology of this condition remains a topic of debate [4]. Delirium is one of the common complications in surgical patients. Postoperative delirium has occurred with a wide range of the incidence rates in surgical units. Hip fracture has a particularly high association with postoperative delirium. The higher incidence after hip fracture surgery may be the result of not only advanced age and more preexisting medical comorbidities but also with the traumatic event [5].

In most studies, risk factors of delirium have mainly been considered in terms of medical and physical aspects [6]. While delirium has been regarded as a result of systemic organic changes, psychological factors have been rarely considered in the occurrence of delirium [7]. This forms a striking contrast to cognitive factors in that cognitive impairment has been reported as a risk factor for delirium in most studies [8]. Some studies reported that preoperative anxiety was a predictive factor for emergence of delirium and major depression was significantly associated with the incidence of delirium [9, 10]. However, these studies did not consider the results with respect to multiple candidate factors and the results were also heterogeneous depending on the variety of collected data. In terms of the relationship with personality, only one report showed that Type D personality, characterized by social inhibition and negative affect, had a nearly significant association with delirium [10].

Although the effect of personality on delirium has not been addressed for a long time, personality traits have been shown to influence health [11]. In particular, a study on the effect of personality on cognitive and psychological aspects has reported that individuals with high neuroticism, who are more likely to experience anxiety, anger, guilt, and depressed mood, showed increased probability of dementia [12]. Hostility, a sub factor of neuroticism, has been suggested to be a strong risk factor for the development of physical diseases such as coronary heart disease [13].

Given the evidence of the contribution of psychological factors on physical health, it is possible that the occurrence of delirium may also be associated with psychological factors. However, there is no prospective systemic study on this issue. In order to define the effects of psychological factors on the occurrence of delirium, we collected psychological data such as anxiety, depression, and personality type before surgery. In this study, we focused on patients with hip fracture because fragility in the setting of mental and physical stress and the higher rate of post-operative delirium in elderly patients may highlight unidentified risk factors for delirium compared with other surgical units. We hypothesized that patients with vulnerable psychological factors would be more likely to develop postoperative delirium. In addition, based on pathophysiological and etiological differences among patients with postoperative delirium, we hypothesized that a data-driven analysis of clinical data using Topological Data Analysis (TDA), which is a recently developed partial clustering method and has been applied in various clinical data [14, 15], would identify meaningful sub-groups of delirium.

## Methods

### Subjects

**Fig. 1:**
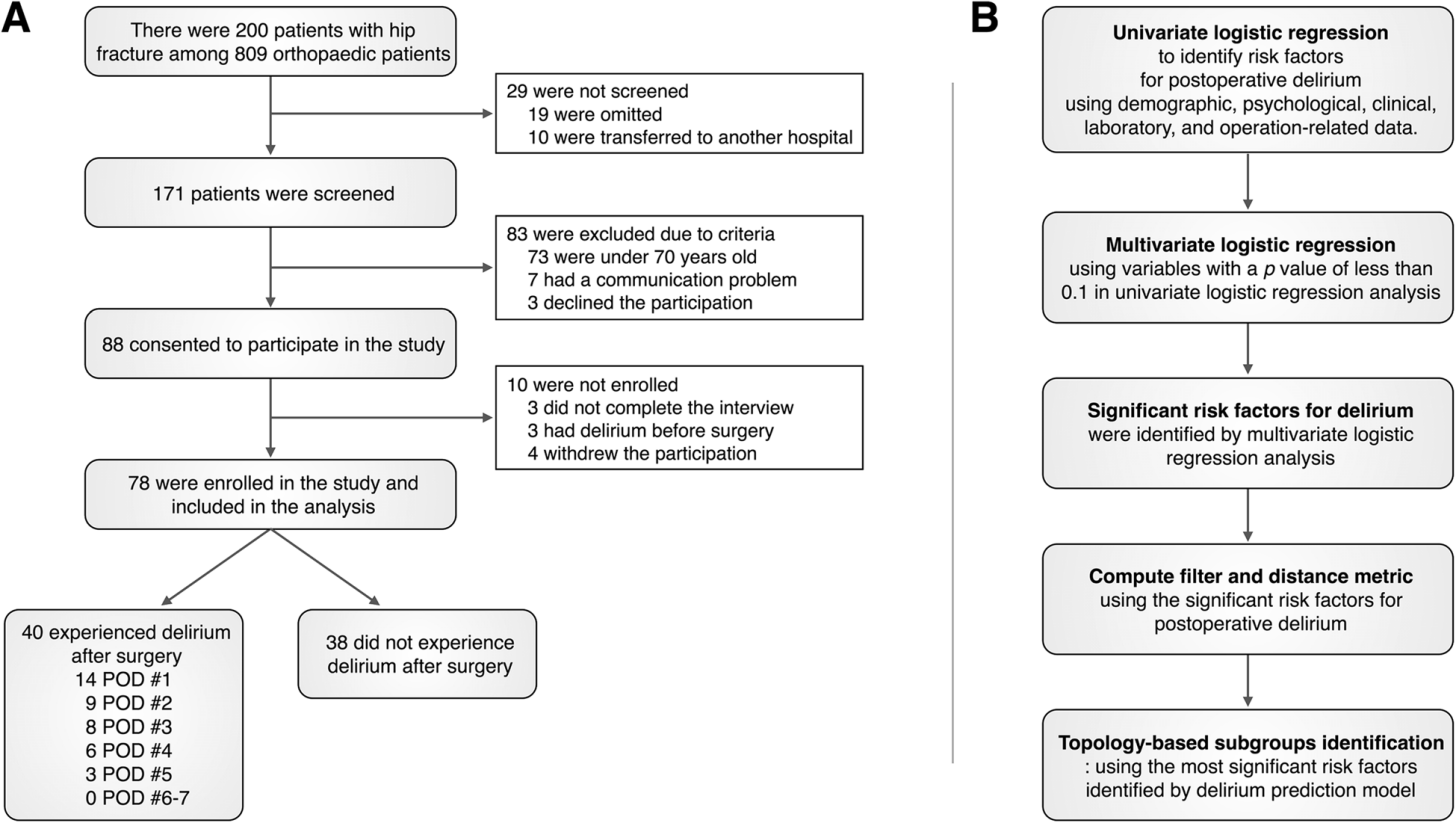
Flowchart for participant enrollment (**a**) and analysis procedures (**b**)

Data were collected under the protocol approved by the Institutional Review Board of Yonsei University Gangnam Severance Hospital. Among 809 orthopedic patients who admitted for the study period of 11 consecutive months in Yonsei University Gangnam Severance Hospital, 200 consecutive patients who fractured their hip due to falling down on the street or in the house and were brought to the emergency room were eligible for the study. There were 29 patients who were not screened due to screening omission and transfer to another hospital. Of the 171 patients with hip fracture, 80 were excluded based on the exclusion criteria such as age < 70 years and too severe communication difficulties to cooperate in the assessment. Additionally, 3 patients declined to participate in this study. After 88 patients provided written informed consent, 10 were not enrolled for various reasons including incomplete interview, delirium before surgery, and withdrawal. The final study sample consisted of the remaining 78 patients (Fig. 1). Emergency surgery was executed on the day they arrived at the emergency room for 9 patients and the next day for the remaining patients.

### Preoperative clinical data collection

Preoperative interview and assessment were done in a supine position in order to prevent the aggravation of pain. The medical history and comorbidities of each patient were obtained from the medical record, and supplemented by patients and a family interview. The medication history about opiates, benzodiazepine, antidepressants, and anticholinergics that may affect postoperative delirium was also collected from the medical record. Cognitive impairment was measured using Mini-Mental State Examination (MMSE), 30-point questionnaire [16]. Preoperative laboratory data including complete blood counts and routine chemistry were collected just after hospitalization. The degree of pain was obtained using Visual Analogue Scale (VAS) when patients were at rest [17]. Opioid administration was recorded, and doses of all opioid drugs were converted to intravenous morphine equivalents using the equianalgesic table [18].

### Preoperative psychological assessment

Psychological scales were collected just after signing a consent to participate in the study. Hamilton Anxiety Scale (HAS) [19] and Hamilton Rating Scale for Depression (HRSD) [20] were used for measuring the severity of anxiety and depression, respectively. The Big Five Inventory (BFI) was used to measure personality traits by asking family members, during which considering the ill state of patients, we used a 10-item short version instead of the 44-item full version [21], which also consists of five facets, including extraversion, agreeableness, neuroticism, conscientiousness, and openness, but has 2-item each facet. This short version proved to retain significant levels of reliability and validity and thus to be useful for research settings with limited time constraints [21]. In addition, to exclude patients with preoperative delirium, a trained psychiatrist evaluated the existence of delirium before patients were transferred to an operating room. Delirium was diagnosed using the Confusion Assessment Method (CAM) [22], which consisted of four items including (a) an acute onset and fluctuating course of mental state, (b) inattention, (c) disorganized thinking, and (d) altered level of consciousness. A diagnosis of delirium satisfied items 1 and 2 essentially and items 3 or 4 selectively.

### Anesthesia and operation

To assess the degree of preoperative illness, an anesthesiologist used the American Society of Anesthesiology Physical Status (ASA PS) classification system with six categories [23], in which higher ASA PS scores indicated more severe preoperative states. The anesthetic method such as regional or general anesthesia was allocated by an anesthesiologist, who decided it based on patients’ ASA PS and his/her clinical preference. Operation procedures included closed reduction and internal fixation, open reduction and internal fixation, or bipolar hemiarthroplasty. Duration of anesthesia was collected in all patients.

### Postoperative assessment of delirium and pain

Every patient was evaluated for delirium around 9 AM in the morning after hip surgery. By a trained psychiatrist, diagnosis of delirium was made using the CAM, and the severity of delirium in cognitive and non-cognitive items was assessed using Korean-Delirium Rating Scale-R-98 (K-DRS) [24]. These measures for delirium were made for 7 days at intervals of 24 h. To measure cognitive decline during delirium, we used the MMSE on the delirium onset day. In addition, everyday throughout the postoperative care period, the degree of pain was obtained again using VAS when patients were at rest, and dose and type of all opioid drugs were recorded.

### Statistical analysis

In order to determine risk factors for postoperative delirium, we performed the univariate logistic regression to select the appropriate candidate variables for multivariate logistic analysis. To find out whether delirium-related characteristics in patients with postoperative delirium were statistically different according to the anesthetic method, the Pearson’s chi-square test was used for the delirium occurrence variable. To confirm whether the difference of case numbers between delirium and non-delirium was statistically significant, we conducted 10,000 random permutation testing. In addition, score test for trend was used for the delirium onset variable, and Student’s *t*-test or Mann-Whitney *U* test was used for continuous data depending on the data distribution.

In order to determine which risk factors contribute to postoperative delirium, we produced a model using logistic analysis. Firstly, univariate logistic analysis was done with each variable as an independent variable and the occurrence of delirium as a dependent variable. Then, we applied the forward selection method in multivariate logistic analysis including any variables with *p* values less than 0.1 in univariate logistic analysis. The final model was produced with variables survived with *p* values less than 0.05 in multivariate logistic analysis. For the appropriateness, Nagelkerke R square and Hosmer-Lemeshow test statistic was used.

### Topology-based data analysis

To describe the patient-patient networks in the dimensions of risk factors, TDA was applied using variables that showed a significant contribution to inferring postoperative delirium. A detailed pipeline for TDA and the mathematical theory underlying TDA have been described in elsewhere [25, 26]. To discover new insight for postoperative delirium, we mapped preoperative clinical variables to the output topology. The Euclidean distance and the variable-normalized first principal component were used as distance metrics for capturing dissimilarity among patients and filter metrics for compressing multi-dimensional features into a single point metric, respectively. Filter metrics were binned into 8 intervals with 80 % overlaps. For each bin of filter metrics, clusters of patients were identified by a single linkage method. Finally, output graph was created where a node indicated a cluster and an edge represented an overlap of patients between any pairs of nodes.

## Results

### Occurrence of postoperative delirium

Postoperative delirium was diagnosed in 40 of 78 patients. As shown in Fig. 1, delirium occurred most commonly in the first postoperative day (*n* = 14; 35.0 %). The occurrence rate decreased with the lapse of time: the second (*n* = 9, 22.5 %), third (*n* = 8, 20.0 %), fourth (*n* = 6, 15.0 %), and fifth (*n* = 3, 7.5 %) postoperative day. There was no patient with delirium that developed on the sixth and seventh day. The duration of delirium ranged from 2 to 33 days (median, 4 day). The mean K-DRS score was 18.97 ± 4.86. Patients with delirium were more likely to have a longer postoperative stay than patients without delirium (median, 10 vs. 9 days; *p* < .05).

### Comparison of the risk factors between patients with and without delirium

Results from univariate analysis which compares the risk factors in patients with and without delirium, are displayed in Table 1. Demographic variables such as age, gender, education level, medical history, addiction factors, and perceptual factors were not associated with delirium. However, patients who medicated with benzodiazepine or antidepressants showed higher probability of postoperative delirium than patients who did not (*p* < .05). Among preoperative assessments, lower MMSE scores were more likely associated with postoperative delirium (*p* < .005), but the preoperative laboratory profiles were not. Among operation-related data, patients under regional anesthesia were significantly more likely to develop delirium (*p* < .05). Other factors, such as ASA PS and the duration of anesthesia, were not statistically significant. Among pain-related data, VAS score and total opioid dose were not associated with delirium regardless of measurement or administration period. In terms of psychological factors, HAS scores and HRSD scores were not significantly associated with the occurrence of delirium. Among the personality traits, extraversion, agreeableness, conscientiousness, and openness were not associated with the risk of delirium, but neuroticism was significantly associated (*p* < .005). Correlation analysis between personality traits and HAD and HRSD scores is presented in Additional file 1: Table S1.

**Table 1:** Patient characteristics and univariate logistic regression for postoperative delirium

	All patients (*n* = 78)	Delirium (*n* = 40)	No Delirium (*n* = 38)	Risk Ratio (95 % CI)	*P*
Demographic data					
General					
Female sex, No. (%)	63 (80.8)	29 (72.5)	34 (89.5)	0.31 (0.09-1.08)	0.07
Age, mean (SD), y	81.6 (6.6)	82.8 (6.2)	80.4 (6.9)	1.06 (0.99-1.13)	0.11
Education, mean (SD), y	8.2 (4.6)	8.7 (3.9)	7.7 (5.1)	1.05 (0.95-1.16)	0.34
Medical history, No. (%)					
Major mental disorder history	13 (16.7)	9 (22.5)	4 (10.5)	2.47 (0.69-8.83)	0.17
Dementia history	19 (24.4)	13 (32.5)	6 (15.8)	2.57(0.86-7.67)	0.09
Delirium history	7 (9.3)	5 (12.8)	2 (5.6)	2.50 (0.45-13.79)	0.29
Other mental disorder history	4 (5.1)	2 (5.0)	2 (5.3)	0.95(0.13-7.09)	0.96
Brain injury history	12 (15.6)	5 (12.5)	7 (18.9)	0.61 (0.18-2.13)	0.44
Psychiatry family history	11 (14.5)	4 (10.3)	7 (18.9)	0.49 (0.13-1.84)	0.29
Hypertension	54 (69.2)	29 (72.5)	25 (65.8)	1.37 (0.52-3.60)	0.52
Diabetes	27 (34.6)	16 (40.0)	11 (28.9)	1.64 (0.64-4.21)	0.31
Addiction history, No. (%)					
Smoking	4 (5.2)	3 (7.7)	1 (2.6)	3.08 (0.31-31.04)	0.34
Alcohol	8 (10.4)	6 (15.4)	2 (5.3)	3.27 (0.62-17.36)	0.16
Preoperative medications, No. (%)					
Opiates	2 (2.6)	0 (0.0)	2 (5.3)	0.00 (-)	-
Benzodiazepines	9 (11.5)	8 (20.0)	1 (2.6)	9.25 (1.10-78.00)	0.04
Antidepressants	11 (14.1)	9 (22.5)	2 (5.3)	5.23 (1.05-26.03)	0.04
Anticholinergics	5 (6.4)	5 (12.5)	0 (0.0)	0.00 (-)	-
Perceptual risk factor, No. (%)					
Visual problem	5 (6.5)	3 (7.5)	2 (5.4)	1.42 (0.22-9.01)	0.71
Auditory problem	12 (15.4)	7 (17.5)	5 (13.2)	1.40 (0.40-4.86)	0.60
Preoperative assessments					
Cognitive ability, mean (SD)					
MMSE	18.7 (6.2)	16.6 (5.7)	20.9 (5.9)	0.88 (0.81-0.96)	0.003
Preoperative laboratory, mean (SD)					
White blood cell count,/μL	9970 (2880)	9810 (2180)	10140 (3500)	0.96 (0.82-1.12)	0.62
Hemoglobin, g/dL	13.1 (11.5)	14.5 (16.0)	11.7 (1.6)	1.09 (0.83-1.44)	0.53
Glucose, mg/dL	149.8 (68.0)	160.7 (79.9)	138.6 (51.8)	1.01 (0.10-1.01)	0.17
Blood Urea Nitrogen, mg/dL	21.1 (8.6)	20.3 (7.7)	21.8 (9.5)	0.98 (0.93-1.03)	0.45
Creatinine, mg/dL	0.8 (0.4)	0.8 (0.2)	0.9 (0.6)	0.58 (0.20-1.74)	0.34
Total protein, g/dL	6.6 (0.5)	6.5 (0.5)	6.7 (0.6)	0.51 (0.21-1.24)	0.14
Albumin, g/dL	3.9 (0.5)	3.8 (0.5)	3.9 (0.5)	0.50 (0.18-0.39)	0.19
Sodium, mEq/L	137.3 (3.8)	137.3 (3.5)	137.3 (4.2)	0.10 (0.89-1.12)	0.96
Potassium, mEq/L	4.2 (0.5)	4.2 (0.4)	4.2 (0.6)	1.02 (0.42-2.48)	0.96
Calcium, mg/dL	8.8 (0.6)	8.7 (0.8)	8.9 (0.6)	0.65 (0.27-1.52)	0.32
Operation-related data					
ASA PS, No. (%)					
2	18 (23.1)	7 (17.5)	11 (28.9)	reference	
3	53 (67.9)	31 (77.5)	22 (57.9)	2.21 (0.74-6.61)	0.15
4	7 (9.0)	2 (5.0)	5 (13.2)	0.63 (0.10-4.18)	0.63
Regional anesthesia, No. (%)	31 (39.7)	21 (52.5)	10 (26.3)	3.10 (1.19-8.02)	0.02
Anesthesia duration, mean (SD), min	123.8 (41.4)	119.8 (38.3)	128.0 (44.6)	0.10 (0.98-1.01)	0.38
Pain-related data					
VAS score, median (IQR)					
At rest before operation	6 (5.0-7.0)	6 (5.0-7.8)	6 (5.0-7.0)	1.08 (0.82-1.42)	0.58
At rest on POD #1	4 (3.0-6.0)	3 (3.0-6.0)	4 (3.0-6.0)	0.85 (0.65-1.11)	0.24
Opioid dose, median (IQR), mg					
Before operation	5 (0-10.0)	5 (0-10.0)	5 (0-9.2)	1.00 (0.95-1.04)	0.84
POD #1	0 (0-6.7)	0 (0-3.3)	0 (0-6.7)	0.92 (0.84-1.01)	0.09
Preoperative psychological scales					
HAS, mean (SD)	6.95 (7.58)	7.97 (7.22)	5.92 (7.89)	1.04 (0.98-1.10)	0.24
HRSD, mean (SD)	4.91 (4.83)	5.55 (4.73)	4.24 (4.91)	1.06 (0.96-1.17)	0.23
Big Five Inventory, mean (SD)					
Extraversion	6.09 (1.66)	5.92 (19.2)	6.27 (1.33)	0.88 (0.67-1.16)	0.36
Agreeableness	6.59 (1.91)	6.36 (2.28)	6.84 (1.40)	0.87 (0.69-1.11)	0.27
Neuroticism	6.99 (1.43)	7.51 (1.47)	6.43 (1.17)	1.86 (1.26-2.78)	0.002
Conscientiousness	7.77 (1.88)	7.41 (1.96)	8.17 (1.73)	0.80 (0.62-1.03)	0.08
Openness	6.27 (1.63)	6.05 (1.87)	6.50 (1.32)	0.84 (0.63-1.12)	0.24

SI conversion factors: To convert white blood cell count to x10^9^/L, multiply values by 0.001; Hemoglobin to g/L, by 10; Glucose and Blood Urea Nitrogen to mmol/L, by 0.0555 and 0.357, respectively; Creatinine to μmol/L, by 88.4; Total protein and Albumin to g/L, by 10; Sodium, Potassium, and Calcium to mmol/L, by 1, 1, and 0.25, respectively

*Abbreviations*: *CI* confidence interval, *SD* standard deviation, *MMSE* mini-mental state examination, *ASA PS* American Society of Anesthesiology Physical Status, *VAS* visual analogue scale, *IQR* interquartile range, *POD* post-operative day, *HAS* hamilton anxiety scale, *HRSD* hamilton rating scale for depression

### Comparison between general and regional anesthesia

As shown in Table 2, delirium developed significantly more in patients who underwent hip surgery under regional anesthesia than general anesthesia (*p* < .05). The onset was also earlier in regional anesthesia than general anesthesia, which was a finding with marginal significance (*p* = .06). On the other hand, the two methods showed no differences in the duration of delirium, K-DRS scores, change of MMSE scores between the delirium onset day and preoperative day, and days of hospitalization (7-36 days for general and 7-45 days for regional). In case of preoperative comorbidity, cerebrovascular disease was more in patients under general anesthesia than in those under regional anesthesia (*p* < 0.05), but there was no group difference in chronic obstructive pulmonary disease, coronary artery related diseases, and chronic renal insufficiency. In regard of preoperative medications and patients’ physical status, both methods also showed no significant difference. As to anesthesia drugs, all patients with postoperative delirium under general anesthesia were given propofol and fentanyl via total intravenous route, while those under regional anesthesia received intravenous bolus shooting of fentanyl and midazolam whenever they were needed. As a result, postoperatively delirious patients with general anesthesia showed more number of fentanyl use (*p* < .01) and more amount of the drug than those with regional anesthesia (*p* < .01). Demographic characteristics and other preoperative information of patients with postoperative delirium according to the anesthetic methods are presented in Additional file 2: Table S2.

**Table 2:** Characteristics of patients with postoperative delirium according to the anesthetic methods

	General anesthesia (*n* = 19)	Regional anesthesia (*n* = 21)	*P*
Occurrence rate, delirium No./total No.	19/47	21/31	0.02^a^
Number of occurrence, No. (%)			0.06^c^
POD 1	5 (26.4)	9 (42.8)	
POD 2	3 (15.8)	6 (28.6)	
POD 3	4 (21.1)	4 (19.0)	
POD 4	5 (26.3)	1 (4.8)	
POD 5	2 (10.5)	1 (4.8)	
POD 6	0 (0.0)	0 (0.0)	
POD 7	0 (0.0)	0 (0.0)	
Onset day, median (min ~ max)	3 (1 ~ 5)	2 (1 ~ 5)	0.07^d^
Days of duration, median (min ~ max)	4 (2 ~ 13)	3 (2 ~ 33)	0.39^d^
Severity: K-DRS scores on the onset day, mean (SD)			
Total	19.8 (4.7)	18.3 (5.0)	0.34^e^
Cognitive	10.8 (2.9)	9.5 (3.2)	0.18^e^
Non-cognitive	8.9 (2.5)	8.8 (2.8)	0.87^e^
Difference in MMSE scores: the onset day minus pre-op day, median (min ~ max)	-6.5 (-25 ~ -3)	-4 (-21 ~ -1)	0.45^d^
Days of hospitalization, median (min ~ max)	11 (7 ~ 36)	9 (7 ~ 45)	0.39^d^
Preoperative comorbidity, No. (%)			
COPD	0 (0.0)	0 (0.0)	-
Cerebrovascular disease	4 (21.1)	0 (0.0)	0.04^b^
CABG/MI/CAD	4 (21.1)	3 (14.3)	0.69^b^
Chronic renal insufficiency	0 (0.0)	0 (0.0)	-
Preoperative medication, No. (%)			
Opiates	0 (0.0)	0 (0.0)	-
Benzodiazepines	3 (15.8)	5 (23.8)	0.70^b^
Antidepressants	6 (31.6)	3 (14.3)	0.27^b^
Anticholinergics	2 (10.5)	3 (14.3)	0.72^b^
Other psychotics	2 (10.5)	1 (4.8)	0.60^b^
ASA PS, No. (%)			0.80^c^
2	3 (15.8)	4 (19.0)	
3	15 (78.9)	16 (76.2)	
4	1 (5.3)	1 (4.8)	
Anesthetic drugs			
Propofol dose, median (min ~ max), mcg/kg/min	11.7 (4.5 ~ 33.3)	-	-
Fentanyl use, No. (%)	19 (100.0)	5 (23.8)	0.00^a^
Fentanyl dose, median (min ~ max), mcg/kg	17.5 (0.5 ~ 22.8)	0.00 (0.00 ~ 2.7)	0.00^d^
Midazolam use, No. (%)	1 (5.3)	2 (9.5)	0.61^b^
Midazolam dose, median (min ~ max), mg/kg	0.00 (0.00 ~ 0.02)	0.00 (0.00 ~ 0.06)	0.83^d^

*Abbreviations*: *POD* postoperative day, *IQR* interquartile range, *COPD* chronic obstructive pulmonary disease, *CABG* coronary artery bypass graft, *MI* myocardial infarction, *CAD* coronary artery disease, *ASA PS* American Society of Anesthesiology Physical Status, *K-DRS* Korean-Delirium Rating Scale-R-98, *SD* standard deviation, *MMSE* mini-mental state examination

^a^Pearson’s chi-square test

^b^Fisher test

^c^score test for trend

^d^Mann-Whitney *U* test

^e^Student *t*-test

To explore the effect of hemodynamic features during regional anesthesia on the occurrence of delirium, the presence of over 20 % reduction in systolic blood pressure or over 90 % reduction in oxygen saturation at baseline was compared between patients with and without delirium, and the variables were not significantly different (odds ratio = 0.406, *p* = .43; odds ratio = 1.313, *p* = .15, respectively). The duration of those events also showed no significant group difference (*x* ^2^ = 0.896, *p* = .40; *x* ^2^ = 2.469 *p* = .21, respectively). Meanwhile, the comparison of preoperative medications and anesthesia-related drugs in patients under regional anesthesia is presented in Additional file 3: Table S3. Both factors showed no significant difference between the presence and absence of postoperative delirium.

### Factors in the final model

The final model from multivariate logistic regression analysis is presented in Table 3. Among variables with a *p* value of less than 0.1 in univariate logistic analysis, variables that remained significant in the forward selection method were narrowed down into lower MMSE score (odds ratio, 0.879; 95 % confidence interval, 0.79-0.98; *p* = .02), regional anesthesia (odds ratio, 3.680; 95 % confidence interval, 1.14-11.89; *p* = .03), higher neuroticism (odds ratio, 2.242; 95 % confidence interval, 1.34-3.75; *p* < .01), and lower conscientiousness (odds ratio, 0.689; 95 % confidence interval, 0.49-0.96; *p* = .03).

**Table 3:** Factors in the final model associated with postoperative delirium

	Regression coefficient	Wald	Risk ratio	*P*	95 % CI for risk ratio
MMSE	-0.129	5.725	0.879	0.02	0.79-0.98
Regional anesthesia	1.303	4.742	3.680	0.03	1.14-11.89
Neuroticism	0.807	9.492	2.242	0.00	1.34-3.75
Conscientiousness	-0.373	4.729	0.689	0.03	0.49-0.96

The model was well fitted with *P* = .15 in Hosmer-Lemeshow goodness-of-fit test, and Nagelkerke R^2^ was 0.427

*Abbreviations*: *CI* confidence interval, *MMSE* mini-mental state examination

### Topology-based subgroup identification

**Fig. 2:**
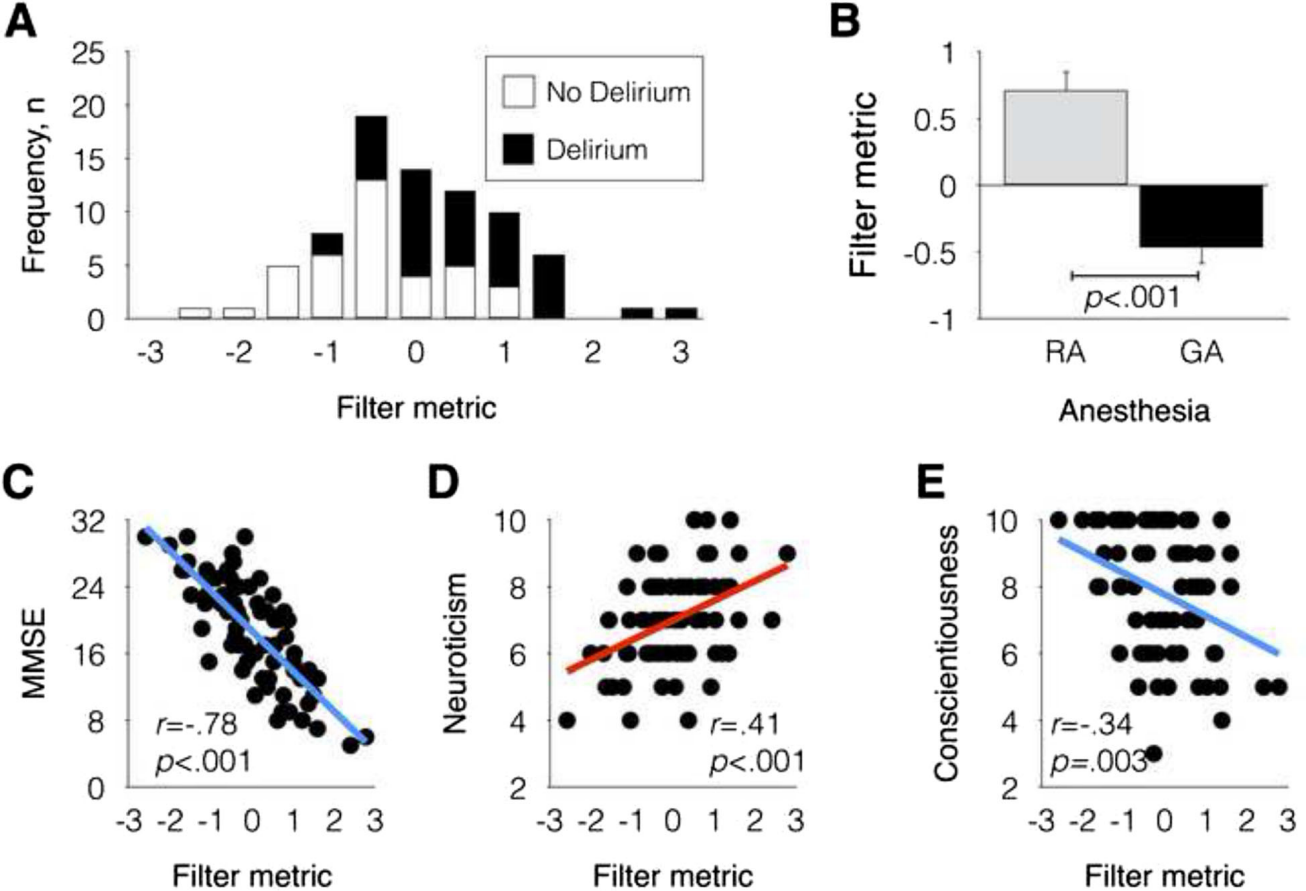
Distribution of filter metric (**a**), average filter metric for general anesthesia (GA) and regional anesthesia (RA) groups (**b**), scatter plot for filter metric and mini mental state examination (MMSE) scores (**c**), scatter plot for filter metric and neuroticism (**d**), and scatter plot for filter metric and conscientiousness (**e**)

**Fig. 3:**
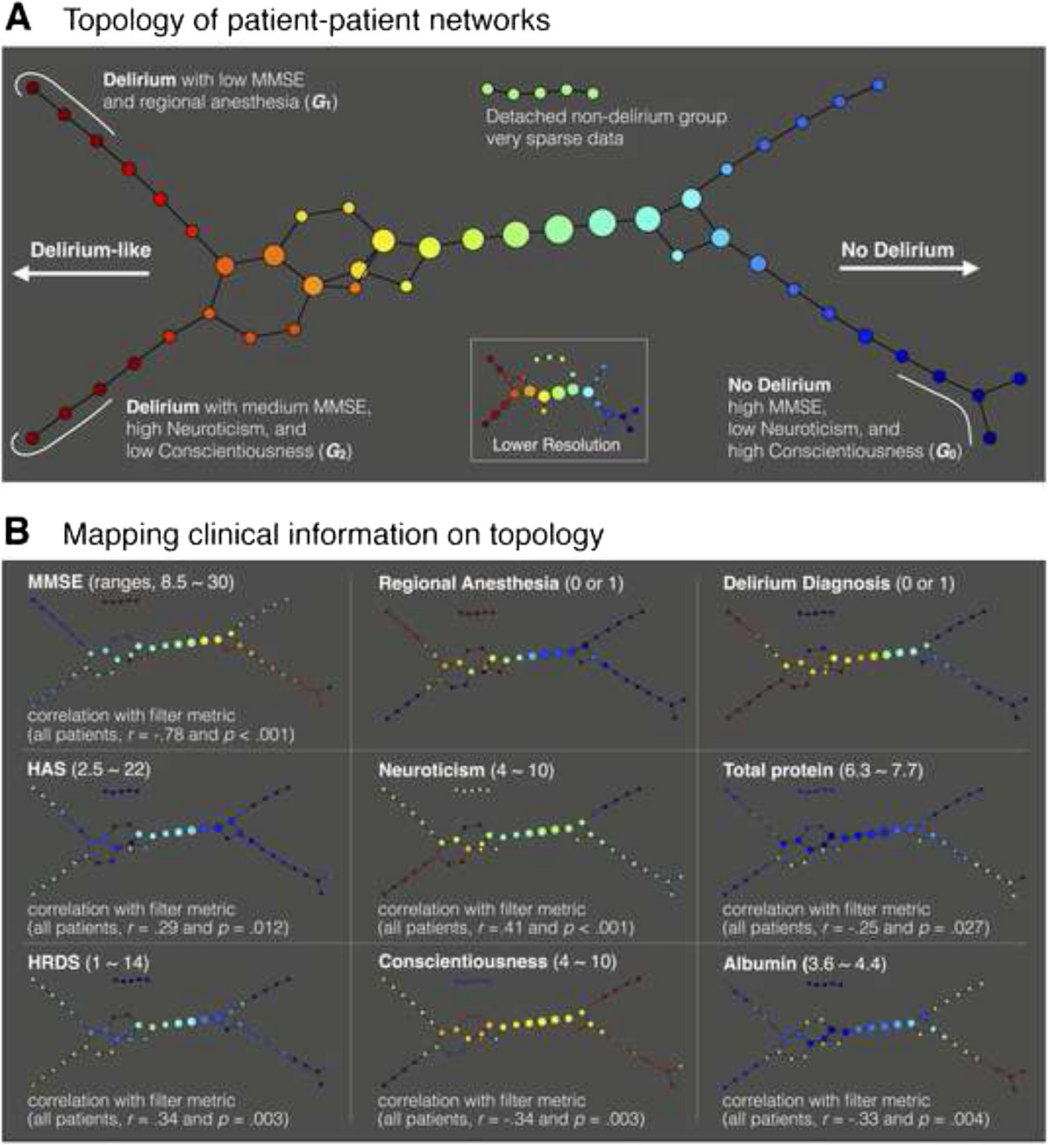
Output of the topological data analysis: **a** Topology of patient-patient networks. Filter metric was subdivided into 8 intervals with 80 % overlap. Several nodes were disconnected from the main graph. An inset graph in bottom right represents a lower resolution topology with 4 intervals and 60 % overlap. A subgroup 1, *G*_*1*_, includes seven delirious patients with a low Mini-Mental State Examination (MMSE) score and regional anesthesia and *G*_*2*_ includes four delirious patients with medium MMSE score, high neuroticism, and low conscientiousness scores. *G*_*0*_ includes six patients with high MMSE, low neuroticism, and high conscientiousness scores; **b** Mapping clinical information on topology. Clinical variables, which showed significant correlations with filter metric, were visualized. The numbers within parenthesis right after the variable name indicate ranges of the node color. Abbreviations: HAS, Hamilton Anxiety Scale; HRDS, Hamilton Rating Scale for Depression

The significant risk factors for postoperative delirium were taken into account for computing filter metrics. Thus, MMSE, neuroticism, conscientiousness, and regional anesthesia were used for evaluating the first principal component. Figure 2 illustrates the distribution and characteristics of filter metric. Given that the first principal component captured 57 % of variance explained, filter metrics contained information from all four input variables and showed a significant negative correlation with MMSE (*r* = -.78, *p* < .001), positive correlation with neuroticism (*r* = .41, *p* < .001), and negative correlation with conscientiousness (*r* = -.34, *p* = .003). Significant differences in filter metrics were also observed between patients with and without delirium (*p* < .001) and between regional and general anesthesia (*p* < .001). Interestingly, filter metrics were inversely correlated with the preoperative laboratory data such as the amount of total protein and albumin (*r* = -.25, *p* = .027; *r* = -.33, *p* = .004, respectively). Furthermore, filter metrics were significantly correlated with HAS and HRSD scores (*r* = .29, *p* = .012; *r* = .34, *p* = .003, respectively). As noted in Fig. 3a and illustrated in Fig. 3b, filter metrics were observed when we divided patients into two groups as a function of anesthesia methods.

TDA detected novel patterns in the patient-patient network, uncovering the complex relationships among MMSE, neuroticism, conscientiousness, and regional anesthesia. As shown in Fig. 3a, TDA produced a flare shape graph with two progressive arms indicating delirium-like patients but different anesthesia methods. We identified two distinct phenotypic subgroups of postoperative delirium in the resulting patient-patient network. The subgroup *G*_*1*_ included seven patients with low MMSE and regional anesthesia (MMSE = 10.7 ± 2.5, neuroticism = 7.3 ± 1.2, conscientiousness = 7.3 ± 2.0, regional anesthesia ratio = 86 %). The subgroup *G*_*2*_ included four delirium patients with medium MMSE, high neuroticism, and low conscientiousness scores (MMSE = 18.25 ± 3.1, neuroticism = 9.5 ± 0.6, conscientiousness = 6.3 ± 2.0, regional anesthesia ratio = 50 %). Patients without delirium having lower filter metrics (bluish nodes in Fig. 3a) were clustered into *G*_*0*_ that included six patients with high MMSE, low neuroticism, and high conscientiousness scores (MMSE = 26.8 ± 2.5, neuroticism = 6.0 ± 0.9, conscientiousness = 9.2 ± 1.0, general anesthesia). Each cluster was associated with distinct nutrition, personality, and cognitive decline variants (see Fig. 3b), which might shed light on the pathophysiology of delirium and drive more precise diagnosis and therapy.

## Discussion

In this study on psychological predictive factors of postoperative delirium, neuroticism and conscientiousness remained a risk factor in the final model. Other factors such as anxiety, depression, and other personality domains were not associated with postoperative delirium according to the logistic regression approach. Overall, we observed that lower MMSE score, higher neuroticism, lower conscientiousness, and regional anesthesia seemed to be related to more frequent occurrence of delirium in patients with advanced age undergoing hip fracture surgery. In addition, topology-based data analysis detected three distinct subgroups of delirium in the dimensions of MMSE, neuroticism, conscientiousness, and anesthesia method.

Among the psychological factors, personality traits which remained significant in the forward selection of multivariate analysis were neuroticism and conscientiousness. Considering that females showed higher conscientiousness and neuroticism than males [27], these two factors might be affected by the gender effect. However, because no significant sex difference in personality traits was observed in our dataset, these two personality factors may be associated with postoperative delirium regardless of the gender effect. In TDA patients with postoperative delirium showed higher neuroticism and lower conscientiousness scores in the patient-patient network. Given that TDA is one of clustering techniques to find hidden patterns or grouping in data but allowing overlaps among clusters [25, 14], a node (or a cluster) in the patient-patient network represents a group of patients with similar patterns of clinical and psychological characteristics and an edge represents the existence of an overlap between two nodes (or clusters). During the time of hospitalization patients with hip fracture may struggle to manage a stressful situation. Unexpected fracture, pain, and surgery as well as being in an unfamiliar hospital setting can be stressors. Therefore, an individual with high neuroticism or low conscientiousness may not be able control himself/herself effectively and may be less likely to cope with a crisis and interpret the situation as threatening and hopelessly difficult. This may be possible because an individual with high neuroticism is more likely to experience anxiety, anger, guilt, and depressed mood [28].

In fact, preoperative anxiety and depression scores were not regarded as a predictive factor of postoperative delirium and were not included in TDA. The contribution of these two psychological factors to delirium has not been conclusive because of conflicting results among previous studies [29]. In the collinearity test, neuroticism was not significantly correlated with anxiety and depression scores though the two scores were correlated each other. Nonetheless, mapping clinical information on topology showed significant positive correlations of filter metrics with HAS and HRSD scores. Filter matric is the first principal component and thus contains all important information of input features. Given that filter metrics were positively correlated neuroticism scores, personality traits related to anxiety and depression rather than these two factors themselves are supposed to cause psychological and physiological changes in patients to develop delirium after surgery.

A mechanism of neuroticism- or conscientiousness-related physical changes has been investigated in terms of the genetic, cellular, and metabolic level. For example, individuals with the top quartile of neuroticism scores or the lowest quartile of conscientiousness scores had a threefold increased risk of Alzheimer’s disease [30]. In metabolic syndrome, neuroticism was associated with its prevalence, whereas individuals who scored in the top 10 % on conscientiousness were 40 % less likely to have it [31]. Neuroticism was identified as a risk factor for heart problems [13, 15]. In addition, neurotransmitter systems that modulate affective states and stress responses were associated with neuroticism scores [32]. Serum cortisol responses to an opioid receptor antagonist were higher in subjects with high neuroticism than with low neuroticism [33]. On the other hand, the conscientiousness element was correlated with cellular immune response [34]. High neuroticism and low conscientiousness were also associated with higher levels of interleukin-6, a pro-inflammatory cytokine [35]. Therefore, neuroticism and conscientiousness may be related to the development of an acute illness such as delirium. Regarding the relationship between stress hormones and systemic inflammation, these personality traits may also be involved in delirium from the genetic to system level via inflammation mechanisms.

Alternatively, neuroticism and conscientiousness may be related to nutritional factors in the development of delirium. According to TDA, the probability of postoperative delirium increased as filter metrics increased. This was not a surprising result because filter metrics were significantly associated with risk factors of postoperative delirium, e.g., the negative correlation with MMSE and conscientiousness scores and positive correlation with neuroticism scores. Furthermore, although levels of serum albumin and total protein were not identified as risk factors of postoperative delirium in logistic regression analysis, they were negatively correlated with filter metric. Considering that reduced protein intake decreases the serum albumin concentration [36, 37] and then contributes to malnutrition and inflammation [38], our results suggest that higher neuroticism and lower conscientiousness scores, which were positively correlated with filter metrics, might contribute to malnutrition in patients with postoperative delirium.

Meanwhile, we found no significant result in other personality traits, including extraversion, agreeableness, and openness. These traits can have a possible link to delirium by influence on the physical state. For example, low agreeableness or high extraversion has been suggested to be associated with drug users [39, 40], and openness is associated with reduced risk of cardiovascular disease [41]. However, because these associations may be limited some specific people, personality factors other than neuroticism and conscientiousness seem to be not important enough to have an effect on the occurrence of postoperative delirium.

In addition to a personality trait, regional anesthesia was associated with postoperative delirium. When we checked the effect of regional anesthesia using TDA, delirious patients with regional and general anesthesia were clearly separated in the patient-patient network by mapping the ratio of regional anesthesia. Studies on the contribution of the anesthetic method to delirium have shown conflicting results. Some studies have postulated that general anesthesia is more frequently associated with delirium because anesthetic agents including propofol influence neuronal processes, leading to changes in neurotransmitter signaling [42]. However, there is also a study reporting no distinct effects on delirium between general and regional anesthesia [43]. Although the difference in the incidence rate of delirium was not statistically significant between the two anesthetic methods, it was higher in regional anesthesia (50 %) than in general anesthesia (38 %) for patients with hip fracture in a prospective study [44]. With regards to detailed information such as delirium severity or onset, there has never been a study according to the anesthetic method. In our study, delirium occurred earlier when regional anesthesia was used compared to general anesthesia. The duration and severity of delirium and cognitive decline during delirium were less in regional anesthesia compared to general anesthesia, though these were not significant. It might be possible that symptoms of delirium, which were more likely to pass unnoticed, may be detected by thorough prospective approaches and result in a higher incidence rate in regional anesthesia. Among possible explanations of regional anesthesia as a risk factor for postoperative delirium, deep sedation levels consistent with general anesthesia were frequently observed during regional anesthesia in elderly patients for hip fracture repair [45]. There was other explanation that a decrease in systolic blood pressure or oxygen saturation during regional anesthesia might be associated with postoperative delirium [46]. However, patients included in our study were not kept under sedation during regional anesthesia and hemodynamic features were not significantly different between patients with and without delirium after regional anesthesia. In the light of these facts, higher incidence of postoperative delirium after regional anesthesia needs to be explained by the effect of psychological factors rather than the complications of this method.

Our results showed that the interactive effects among regional anesthesia, neuroticism, and conscientiousness were associated with subgroups of postoperative delirium as observed in the patient-patient network (see insets in the second column of Fig. 3b). TDA provided more intuitive information by mapping personality traits and a ratio of regional anesthesia as a color in the node. Herein, it may be important to pay attention to the characteristics of ‘hip fracture repairs.’ During regional anesthesia, patients are usually in a voice-reacting conscious state [47], and thus can respond to some stimuli. Specifically, the operative setting in an orthopedic unit such as being undressed around unfamiliar peoples, surgical positioning, unfamiliar environment, and sounds of a drill or hammer during surgery may cause stress in many patients. These uncomfortable experiences may provoke psychological disturbances, particularly in patients with high neuroticism. Taken together, regional anesthesia in an orthopedic unit may cause postoperative delirium by placing the patients in an environment that affects psychological aspects. In this sense, the implications of our results for patients with hip fracture are that personality traits should be considered when the anesthetic method is chosen and the environment should be optimized for a less stimulating setting when regional anesthesia is used.

Meanwhile, MMSE score remained a risk factor in the final model even after confounding factors were added for correction. Although the statistical power was somewhat low, there is no doubt that preoperative cognitive ability was associated with the occurrence of postoperative delirium. This finding is consistent with previous reports that cognitive impairment is one of the risk factors for delirium [1, 8]. Although MMSE score and neuroticism proved to be an independent risk factor, it should be considered that cognitive ability including attention, perception, memory, problem solving, and comprehension may affect psychological factors such as anxiety and depression [48]. In addition, most other studies have reported that advanced age is a risk factor for delirium [1]. However, age itself was not a significant factor in our study and had marginal significance with a *p*-value of 0.07 in univariate analysis. This may be due to the fact that age in our inclusion criteria (aged ≥ 70) was relatively high and the variation of age was relatively small. Previous studies have also documented that pain or inadequate pain control is likely to increase the risk of delirium [49], whereas our study showed that the degree of pain and the following opioid medication were not associated with the occurrence of postoperative delirium.

There are some limitations in this study. First, our data were collected within a restricted period in only one center which resulted in a relatively small size for a prospective study. Although TDA produced informative outputs regarding personality traits and anesthesia types, identification of clinically meaningful subgroups of postoperative delirium was not achieved due to a small sample size. Second, although TDA has an advantage in deeper understanding of a disease, its application has been limited in several medical diseases such as diabetes, breast cancer [50], and attention deficit hyperactivity disorder [51]. More studies are necessary to determine its usefulness in the clinical domain. Third, more comprehensive psychological assessments were not performed because all patients were acutely ill due to hip fracture and other traumatic injuries. Because of the same reason, personality traits were measured using the short version instead of the full version of the BFI. However, this short version may be rather useful to acutely ill patients because it was proved to be reliable and valid [21]. Forth, because patients were acutely ill and preoperative assessment was done in a supine position in order to prevent the aggravation of pain, their baseline MMSE scores seemed to be underscored.

## Conclusions

Our results showed that personality traits of neuroticism and conscientiousness were important risk factors for postoperative delirium and, particularly, the influence of personality traits was increased under regional anesthesia. This study verifies the contribution of psychological risk factors to delirium and provides new insight for complex etiologies of delirium by mapping various clinical variables in the topological space. Our study suggests that mapping patients’ psychological features in the topological space need to be considered before deciding an anesthetic method. It may be feasible in that the short version of BFI can be easily measured by asking family members before the operation. Prospective data gathering in our study might arouse attention to the occurrence of delirium and thus lead to more appropriate management. A future study with increased sample size and more comprehensive psychological assessments is needed to achieve a comprehensive understanding of postoperative delirium and translate TDA results into clinics to provide a group-specific treatment or personalized medicine.

## Additional files

Additional file 1: Table S1. Correlation coefficients between personality traits of the Big Five Inventory and anxiety and depression scales. (DOCX 15 kb)
Additional file 2: Table S2. Demographic characteristics of patients with postoperative delirium according to the anesthetic methods. (DOCX 18 kb)
Additional file 3: Table S3. Characteristics of patients under regional anesthesia according to postoperative delirium. (DOCX 16 kb)
