# Supplementary material for: A personality trait contributes to the occurrence of postoperative delirium: a prospective study
Source: BMC Psychiatry. 2016 Nov 3;16:371. doi: 10.1186/s12888-016-1079-z (PMC5094033; doi:10.1186/s12888-016-1079-z)
Supplement: Additional file 1: Table S1. — Correlation coefficients between personality traits of the Big Five Inventory and anxiety and depression scales. (DOCX 15 kb) [file 12888_2016_1079_MOESM1_ESM.docx]

**Supplementary table 1.**  Correlation coefficients between personality traits of the Big Five Inventory and anxiety and depression scales

|  | All patients  (n=78) | | Delirium  (n=40) | | No Delirium  (n=38) | |
| --- | --- | --- | --- | --- | --- | --- |
|  | HAS | HRSD | HAS | HRSD | HAS | HRSD |
| Extraversion | 0.04 | -0.06 | 0.11 | -0.16 | 0.00 | 0.12 |
| Agreeableness | -0.13 | -0.10 | -0.17 | -0.70 | -0.07 | -0.11 |
| Neuroticism | 0.11 | 0.10 | 0.21 | 0.17 | -0.08 | -0.07 |
| Conscientiousness | -0.08 | -0.30^**^ | -0.18 | -0.42^**^ | 0.08 | -0.13 |
| Openness | -0.17 | -0.19 | -0.07 | -0.03 | -0.27 | -0.39^*^ |

Abbreviations: HAS, Hamilton Anxiety Scale; HRSD, Hamilton Rating Scale for Depression

^*^ *p* < 0.05; ^**^ *p* < 0.01
