# Supplementary material for: A personality trait contributes to the occurrence of postoperative delirium: a prospective study
Source: BMC Psychiatry. 2016 Nov 3;16:371. doi: 10.1186/s12888-016-1079-z (PMC5094033; doi:10.1186/s12888-016-1079-z)
Supplement: Additional file 2: Table S2. — Demographic characteristics of patients with postoperative delirium according to the anesthetic methods. (DOCX 18 kb) [file 12888_2016_1079_MOESM2_ESM.docx]

**Supplementary table 2.**  Demographic characteristics of patients with postoperative delirium according to the anesthetic methods

|  | General anesthesia  (n=19) | Regional anesthesia  (n=21) | P |
| --- | --- | --- | --- |
| Demographic data |  |  |  |
| General |  |  |  |
| Female sex, No. (%) | 14 (73.7) | 15 (71.4) | 0.87^a^ |
| Age, mean (SD) | 81.1 (6.2) | 84.2 (5.8) | 0.11^b^ |
| Education years, median (min~max) | 9 (0~16) | 9 (6~16) | 0.80^c^ |
| Medical history, No. (%) |  |  |  |
| Major mental disorder history | 4 (21.1) | 5 (23.8) | 1.00^d^ |
| Dementia history | 4 (21.1) | 9 (42.9) | 0.14^a^ |
| Delirium history | 3 (16.7) | 2 (9.5) | 0.65^d^ |
| Other mental disorder history | 1 (5.3) | 1 (4.8) | 1.00^d^ |
| Brain injury history | 5 (26.3) | 0 (0.0) | 0.02^d^ |
| Psychiatry family history | 5 (26.3) | 8 (38.1) | 0.43^a^ |
| Hypertension | 16 (84.2) | 13 (61.9) | 0.12^a^ |
| Diabetes | 11 (57.9) | 5 (23.8) | 0.03^a^ |
| Addiction history, No. (%) |  |  |  |
| Smoking | 2 (11.1) | 1 (4.8) | 0.59^d^ |
| Alcohol | 2 (11.1) | 4 (19.0) | 0.67^d^ |
| Perceptual risk factor, No. (%) |  |  |  |
| Visual problem | 0 (0.0) | 3 (14.3) | 0.23^d^ |
| Auditory problem | 2 (10.5) | 5 (23.8) | 0.41^d^ |
| Pain-related data |  |  |  |
| VAS score, median (min~max) |  |  |  |
| At rest before operation | 7 (3~10) | 6 (2~8) | 0.19^c^ |
| At rest on POD #1 | 3 (3~8) | 3 (2~8) | 0.19^c^ |
| Opioid dose, median (min~max), mg |  |  |  |
| Before operation | 0.0 (0.0~15.0) | 5.0 (0.0~53.0) | 0.31^c^ |
| POD #1 | 0.0 (0.0~16.7) | 0.0 (0.0~6.7) | 0.18^c^ |
| Preoperative assessments |  |  |  |
| MMSE | 17.3 (4.9) | 16.0 (6.3) | 0.47^b^ |
| HAS, median (min~max) | 7 (0~25) | 7.5 (0~23) | .88^c^ |
| HRSD, median (min~max) | 5 (0~16) | 5 (0~14) | .91^c^ |

Abbreviations: SD, standard deviation; VAS, Visual Analogue Scale; POD, post-operative day; MMSE, Mini-Mental State Examination; HAS, Hamilton Anxiety Scale; HRSD, Hamilton Rating Scale for Depression

^a^ Pearson’s chi-square test

^b^ Student t-test

^c^ Mann-Whitney U test

^d^ Fisher test
