# Supplementary material for: A personality trait contributes to the occurrence of postoperative delirium: a prospective study
Source: BMC Psychiatry. 2016 Nov 3;16:371. doi: 10.1186/s12888-016-1079-z (PMC5094033; doi:10.1186/s12888-016-1079-z)
Supplement: Additional file 3: Table S3. — Characteristics of patients under regional anesthesia according to postoperative delirium. (DOCX 16 kb) [file 12888_2016_1079_MOESM3_ESM.docx]

**Supplementary table 3.**  Characteristics of patients under regional anesthesia according to postoperative delirium

|  | Delirium  (n=21) | No Delirium  (n=10) | P |
| --- | --- | --- | --- |
| Preoperative medication, No. (%) |  |  |  |
| Opiates | 0 (0.0) | 0 (0.0) | - |
| Benzodiazepines | 5 (23.8) | 0 (0.0) | 0.15^a^ |
| Antidepressants | 3 (14.3) | 1 (10.0) | 1.00^a^ |
| Anticholinergics | 3 (14.3) | 0 (0.0) | 0.53^a^ |
| Other psychotics | 1 (4.8) | 0 (0.0) | 1.00^a^ |
| Anesthesia characteristics |  |  |  |
| Fentanyl use, No. (%) | 5 (23.8) | 6 (60.0) | 0.11^a^ |
| Fentanyl dose, median (min~max), mcg/kg | 0.00 (0.00~2.7) | 0.76 (0.00~41.1) | 0.07^b^ |
| Midazolam use, No. (%) | 2 (9.5) | 3 (30.0) | 0.30^a^ |
| Midazolam dose, median (min~max), mg/kg | 0.00 (0.00~0.06) | 0.00 (0.00~0.06) | 0.25^b^ |

^a^ Fisher test

^b^ Mann-Whitney U test
